# Supplementary material for: Disorder-dominated quantum criticality in moiré bilayers
Source: Nat Commun. 2022 Dec 3;13:7469. doi: 10.1038/s41467-022-35103-w (PMC9719564; doi:10.1038/s41467-022-35103-w)
Supplement: Supplementary file 1 — Supplementary Information [file 41467_2022_35103_MOESM1_ESM.pdf]

Supplementary Information for Disorder-dominated quantum criticality in moiré bilayers

Yuting Tan,[1*] Pak Ki Henry Tsang,[1]
Vladimir Dobrosavljević[1†]

[1]Department of Physics and National High Magnetic Field Laboratory,
Florida State University, Tallahassee, Florida 32306, USA

*E-mail: ytan@magnet.fsu.edu; †E-mail: vlad@magnet.fsu.edu.

Supplementary Note 1. Models and Methods

Model and lattice

In this section, we provide details on the theoretical framework applied in the main manuscript. As shown in the main text, we study the following class of Hamiltonians combining disorder and electron-boson interactions on a triangular lattice:

$$\begin{aligned}\mathcal{H} = & - t\sum_{\langle i,j\rangle} c_i^\dagger c_j + \sum_{i\in A}\epsilon_A c_i^\dagger c_i + \sum_{i\in B}\epsilon_B c_i^\dagger c_i \\ & + \sum_i(\xi_i-\mu)c_i^\dagger c_i + g\sum_i X_i(c_i^\dagger c_i - n) + H_b,\end{aligned} \tag{S1}$$

The Brillouin zone in k-space of the triangular lattice is shown in Supplementary Fig. 1a. The bare band structure (without coupling to disorder and bosons) for triangular lattice can be written as:

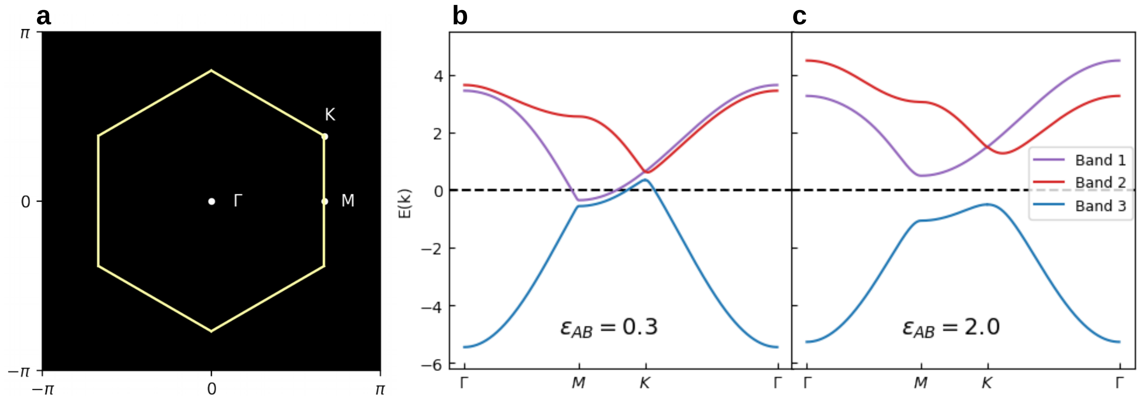

Supplementary Figure 1: Triangular lattice. **a** Brillouin zone in k-space; **b**, **c** Band structures for band splitting $\epsilon_{AB} = 0.3, 2.0$ correspondingly, without disorder and interactions.

$$\mathbf{E}\left(\mathbf{k}, \epsilon_A, \epsilon_B\right) = \begin{bmatrix} \epsilon_A & 0 & 0 \\ 0 & \epsilon_A & 0 \\ 0 & 0 & \epsilon_B \end{bmatrix} + \boldsymbol{\epsilon}\left(\mathbf{k}\right) = \begin{bmatrix} \epsilon_A & E_{12} & E_{13} \\ E_{12}^* & \epsilon_A & E_{23} \\ E_{13}^* & E_{23}^* & \epsilon_B \end{bmatrix} \tag{S2}$$

where

$$\begin{aligned} E_{12} &= -t\left(e^{ik\delta_{ab}^{(1)}} + e^{ik\delta_{ab}^{(2)}} + e^{ik\delta_{ab}^{(3)}}\right); \\ E_{13} &= -t\left(e^{ik\delta_{ac}^{(1)}} + e^{ik\delta_{ac}^{(2)}} + e^{ik\delta_{ac}^{(3)}}\right); \\ E_{23} &= -t\left(e^{ik\delta_{bc}^{(1)}} + e^{ik\delta_{bc}^{(2)}} + e^{ik\delta_{bc}^{(3)}}\right); \end{aligned} \tag{S3}$$

and

$$\begin{aligned} \delta_{ac}^{(1)} &= (a, 0)\,, \quad \delta_{ac}^{(2)} = \left(-\frac{a}{2}, \frac{\sqrt{3}a}{2}\right), \delta_{ac}^{(3)} = \left(-\frac{a}{2}, -\frac{\sqrt{3}a}{2}\right), \\ \delta_{ab}^{(1)} = \delta_{bc}^{(1)} &= (-a, 0)\,, \delta_{ab}^{(2)} = \delta_{bc}^{(2)} = \left(\frac{a}{2}, \frac{\sqrt{3}a}{2}\right), \quad \delta_{ab}^{(3)} = \delta_{bc}^{(3)} = \left(\frac{a}{2}, -\frac{\sqrt{3}a}{2}\right), \end{aligned} \tag{S4}$$

and a is the lattice spacing. After diagonalizing the matrix in Eq. S2, we can plot the bare band structure with different band splitting, as shown in Supplementary Fig. 1b and 1c, which just reflect the bare band transition.

Mean field approach: CPA-DMFT

We solve the model on CPA-DMFT level (*1, 2*). The local Green function is

$$\tilde{\mathbf{G}}_{\mathbf{O}}\left(\omega\right)=\left[(\omega+\mu)I-\left[\begin{array}{ccc}\epsilon_A+\Sigma_A & 0 & 0\\ 0 & \epsilon_A+\Sigma_A & 0\\ 0 & 0 & \epsilon_B+\Sigma_B\end{array}\right]-\boldsymbol{\Delta}_{\mathbf{O}}(\omega)\right]^{-1} \tag{S5}$$

where $\tilde{\mathbf{G}}_{\mathbf{O}}\left(\omega\right)$ and hybridization function $\boldsymbol{\Delta}_{\mathbf{O}}(\omega)$ are diagonal. The green function for the lattice is

$$\mathbf{G}\left(\omega,\mathbf{k}\right)=\left[\left(\omega+i\eta+\mu\right)\hat{I}-\left[\begin{array}{ccc}\epsilon_A+\Sigma_A & 0 & 0\\ 0 & \epsilon_A+\Sigma_A & 0\\ 0 & 0 & \epsilon_B+\Sigma_B\end{array}\right]-\boldsymbol{\epsilon}\left(\mathbf{k}\right)\right]^{-1} \tag{S6}$$

The AA(BB) element of $\tilde{\mathbf{G}}_{\mathbf{O}}\left(\omega\right)$ is

$$\tilde{G}_{\mathbf{O}}^{AA(BB)}\left(\omega,\epsilon_A,\epsilon_B\right)=\sum_{\mathbf{k}}\mathbf{G}\left(\omega,\mathbf{k}\right)_{AA(BB)} \tag{S7}$$

From Eqs. S5$\sim$S7 we obtain the hybridization function as

$$\Delta_{\mathbf{O}}^{AA(BB)}\left(\omega\right)=\omega+i\eta+\mu-\epsilon_{A(B)}-\Sigma_{A(B)}-\left[\tilde{G}_{\mathbf{O}}^{AA(BB)}\right]^{-1} \tag{S8}$$

With specific disorder ξ and boson displacement X on site A(B), the green function can be written as

$$G_{A(B)}\left(\omega,\xi,X\right)=\left[\omega+i\eta+\mu-\epsilon_{A(B)}-\xi-gX-\Delta_{\mathbf{O}}^{AA(BB)}\left(\omega\right)\right]^{-1} \tag{S9}$$

By averaging this Green function with disorder and bosons, we again obtain the local green function:

$$\tilde{G}_{\mathbf{O}}^{AA(BB)}\left(\omega\right)=\int d\xi\int dX P_0\left(\xi\right)P_{A(B)}\left(\xi,X\right)G_{A(B)}\left(\omega,\xi,X\right), \tag{S10}$$

with the boson probability distribution:

$$\begin{aligned}P_{A(B)}\left(\xi,X\right) &= \frac{1}{Z_{loc}\left(\xi\right)}\exp\left\{\frac{1}{T}\left\{-\frac{X^2}{2}+gXn\right.\right.\\ &\left.\left.+\int_{-\infty}^{\infty}d\omega f\left(\omega,T\right)\left[-\frac{1}{\pi}\Im\left[\ln\left(-G_{A(B)}^{-1}\left(\omega,\xi,X\right)\right)\right]\right]\right\}\right\}\end{aligned} \tag{S11}$$

with $Z_{loc}(\xi) = \int_{-\infty}^{\infty} dX P_{A(B)}(\xi, X)$, the Fermi function $f(\omega, T) = 1/[1 + \exp(\omega/T)]$ and the occupation number per site

$$n = \frac{1}{3}\int_{-\infty}^{\infty} d\omega f(\omega, T)\left\{-\frac{1}{\pi}\Im\left[\mathrm{Tr}\tilde{\mathbf{G}}_{\mathbf{O}}(\omega)\right]\right\}. \tag{S12}$$

These equations form a complete self-consistent loop for this model. The chemical potential is adjusted so that $n = 1/3$ with 10^{-6} accuracy.

The DC conductivity is calculated via Kubo formula (*3*).:

$$\sigma_{\mu\nu} = \frac{2e^2}{\pi\Omega}\int d\omega[-f'(\omega)]\sum_{\mathbf{k}}\mathrm{Tr}\left\{\frac{\partial\mathbf{E}(\mathbf{k})}{\partial k_\mu}\Im\mathbf{G}(\omega,\mathbf{k})\frac{\partial\mathbf{E}(\mathbf{k})}{\partial k_\nu}\Im\mathbf{G}(\omega,\mathbf{k})\right\}. \tag{S13}$$

with

$$\begin{aligned}\mathbf{v}_\mu(\mathbf{k}) &= \frac{\partial\mathbf{E}(\mathbf{k})}{\partial k_\mu} \\ &= \begin{bmatrix} 0 & -it\sum_n\left(\delta_{ab}^{(n)}\right)_\mu e^{ik\delta_{ab}^{(n)}} & -it\sum_n\left(\delta_{ab}^{(n)}\right)_\mu e^{ik\delta_{ac}^{(n)}} \\ it\sum_n\left(\delta_{ab}^{(n)}\right)_\mu e^{-ik\delta_{ab}^{(n)}} & 0 & -it\sum_n\left(\delta_{ab}^{(n)}\right)_\mu e^{ik\delta_{bc}^{(n)}} \\ it\sum_n\left(\delta_{ab}^{(n)}\right)_\mu e^{-ik\delta_{ac}^{(n)}} & it\sum_n\left(\delta_{ab}^{(n)}\right)_\mu e^{-ik\delta_{bc}^{(n)}} & 0 \end{bmatrix}\end{aligned} \tag{S14}$$

We only focus on the xx component of the conductivity.

As any mean-field theory, CPA-DMFT cannot be expected to accurately describe the immediate vicinity of the critical point. However, again as any standard mean-field theories, it should work well over a broad parameter range surrounding the transition – precisely as in the current experiments. Better understanding the narrow "asymptotic" regime remains an interesting question for future work. This particular set of equations we are presenting here can be obtained, as it turns out, as a saddle point approximation to a certain field theory, which in principle allows systematic corrections to mean field theory. Work along these lines is in progress.

We should mention that although our CPA-DMFT setup can - in principle - describe different forms/types of disorder, its form generally suffers significant renormalizations close to the transition. Due to certain polaronic effects (*1*), any smooth distribution of disorder ends up

renormalizing to a bimodal (binary-like) form, facilitating the opening of a spectral gap at the Fermi energy at any filling. This effect, which is a robust feature, at least within our DMFT-type mean-field formulation, suggests that the results should be quite robust to the precise form of bare disorder.

Supplementary Note 2. Theoretical Results

Fermi surface and the increasing A, the slope of $R(T)$

We calculate the Fermi surface (Supplementary Fig. 2) as the MIT is approached. We can see clearly that the number of available carriers (or Fermi energy) decreases as the size of the electron (hole) Fermi pockets shrinks. So the relative electron-boson coupling increases, which leads to the initial increase of the slope of $R(T)$ at low T in the metallic phase.

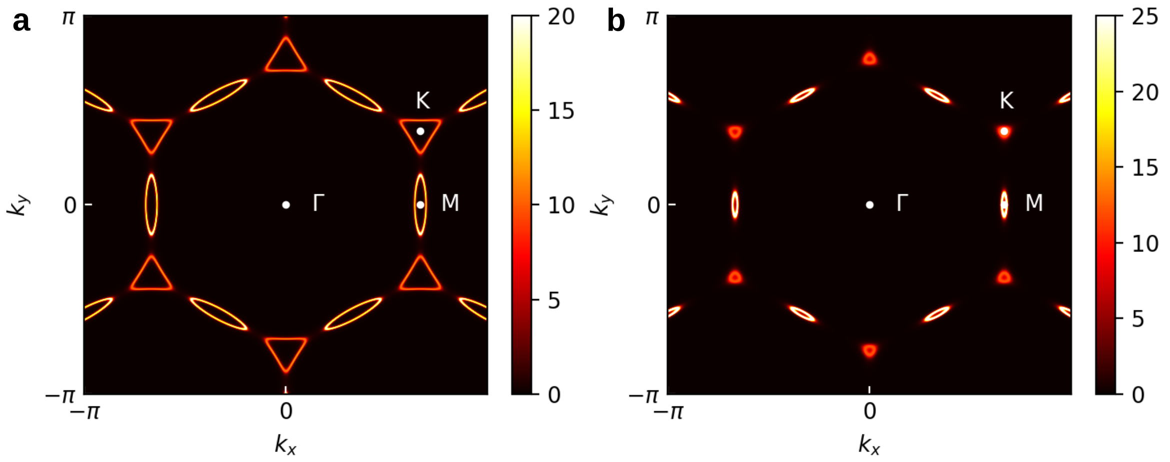

Supplementary Figure 2: Colors on plot are the spectral function at the Fermi energy: $A(\omega = 0, \mathbf{k}) = -\frac{1}{\pi}\Im\left[\mathrm{Tr}\mathbf{G}\left(\omega = 0, \mathbf{k}\right)\right]$. These are the Fermi surfaces for band splitting **a** $\epsilon_{AB} = 0.4$, **b** $\epsilon_{AB} = 0.8$, with $W = 1, g = 1, T = 0.004$.

Intrinsic nature of the Metal-Insulator Transition (MIT)

Referring to the intrinsic nature of this MIT, as shown in the ARPES plot (Supplementary Fig. 3) for the spectral function: $A(\omega, \mathbf{k}) = -\frac{1}{\pi}\Im\left[\mathrm{Tr}\mathbf{G}\left(\omega, \mathbf{k}\right)\right]$, before the MIT, we can not distinguish

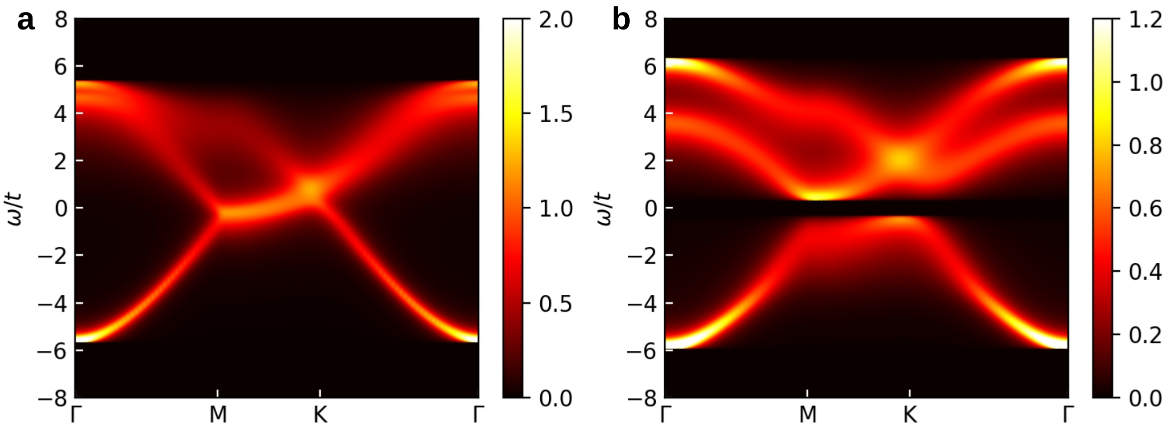

Supplementary Figure 3: Spectral function for band splitting a $\epsilon_{AB} = 0.8$, **b** $\epsilon_{AB} = 3.2$, with $W = 4, g = 1, T = 0.008$

bands in present of disorder (Supplementary Fig. 3a), although we can still find sharp band edges. So we just have one band and two band edges in this case. After MIT, a hard gap in density of state (DOS) shows up (Supplementary Fig. 3b), so we have four sharp band edges and two bands. The number of bands is changing in this CPA type of transition, which makes the nature of this transition totally different from the bare band transition, in which the number of bands is conserved.

In addition, in this CPA transition, as shown in Supplementary Fig. 4, the density of state per site $\rho(\omega) \sim \omega^{1/3}$ at this very low T (the effect of T can be neglected.) so the critical exponent for ρ is $1/3$, as in other CPA transitions (unpublished work by the authors). Without the loss of generality, a slightly larger level of disorder ($W = 4$) was used in these plots for comparing the blurred bands and the sharp band edge, and also to obtain a larger size of the critical region, so that we can see the critical behavior of ρ more clearly.

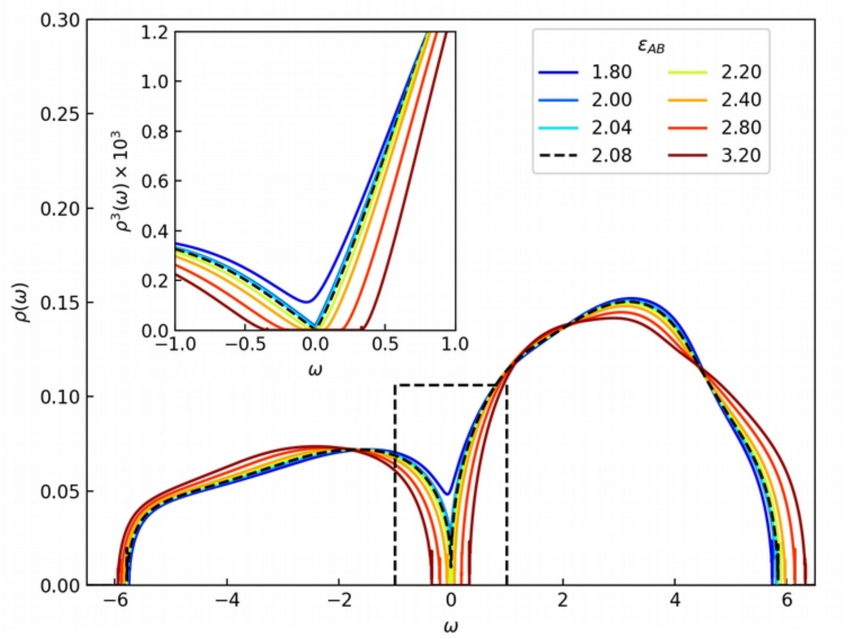

Supplementary Figure 4: DOS per site $\rho(\omega)$ for varies ϵ_{AB}, with $W = 4, g = 1, T = 0.008$. The black dashed line corresponds to critical ϵ_{AB}. $\rho \sim \omega^{1/3}$ at the very low temperature T as shown in the inset. So the critical power of ρ is just $1/3$, as in CPA transition.

Supplementary Note 3. Detailed analyses of characteristic properties: A, T^* and Δ

In this section, we present the main information for the detailed analysis to the theoretical results. The analyses for the experiments are done in an identical fashion. In the insulating phase, one would expect activation behavior for resistance vs temperature: $R(T) \sim \exp(\Delta/T)$, when $T < \Delta$. Following the same procedures in experiment paper (*4*), we plot Ln(R) vs $1/T$ for our theoretical results (Supplementary Fig. 5b), and extract the slope as the activation gap Δ. On the metallic side, the resistance curves display linear-T behavior at low temperatures:$R(T) \approx R_o + AT$. We fit the curves linearly (Supplementary Fig. 5a) at low temperatures and from that, we can extract the slope A, R_o, as well as the characteristic temperature T^*, which is determined by eyeballing the position where the curves start to deviate from the linear trend.

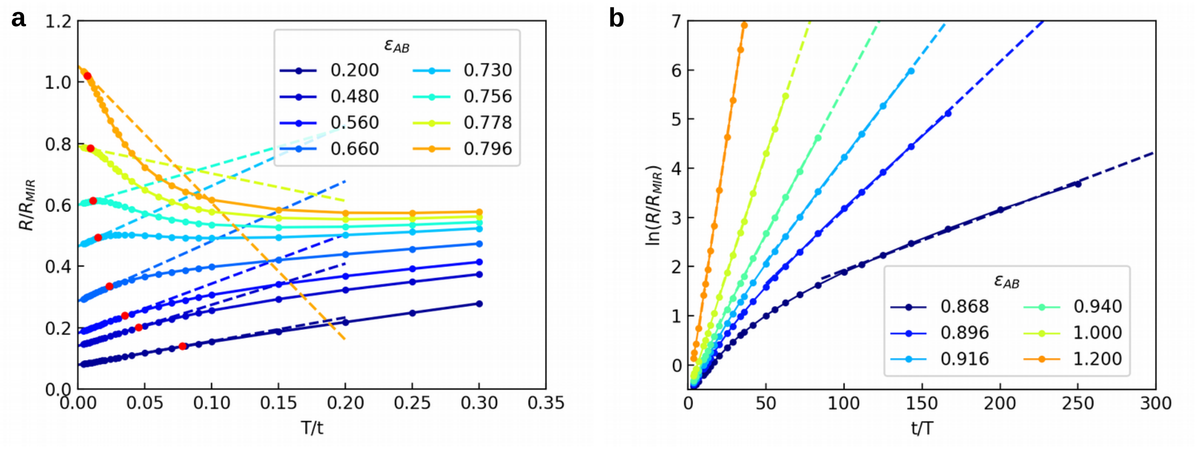

Supplementary Figure 5: Illustration of obtaining three characteristic properties, a, A**,** T^* **and b,** Δ **from the resistance curves. a** We linearly fit the resistance curves at low Ts (dashed lines), obtaining the slope A and T^* (red dots), and the latter is determined by eyeballing the position where the curves start to deviate from the linear trend. **b** On the insulating side, the activation gap Δ is obtained from linear fitting $\text{Ln}(R) \sim 1/T$ at low Ts (slope of the dashed lines), where it displays activation behavior. Same analyses are performed on the experimental results.

Supplementary Note 4. Analyses for data obtained from Device 1

The manuscript focus on the experimental data obtained from Device 2. We also perform the same analyses on Device 1 (*4*), as shown in Supplementary Fig. 6 and Supplementary Fig. 7. Remarkably, all the quality and some quantity trends are the same. The critical exponent $z\nu = 0.9 \pm 0.1$, which is also very close to 1. We also compare the Log-linear plot for resistance curves for Device 2, Device 1, and the theory in Supplementary Fig. 8, which display excellent agreement. We can also compare the disorder strength in both devices by examining the residual ($T = 0$) resistivity in the metallic regime. The ratio of the high-T resistivity and the residual resistivity is typically used to quantify the amount of disorder in a given material, because in metals the high-T resistivity is dominated by thermal scattering (often due to phonons) and not by disorder, while the residual resistivity is controlled (mostly) by disorder. For Device 2, the ratio $R_{T=50K}/R_{T=1.6K}$ of the most metallic curve is around 5, while for Device 1 it is around 8,

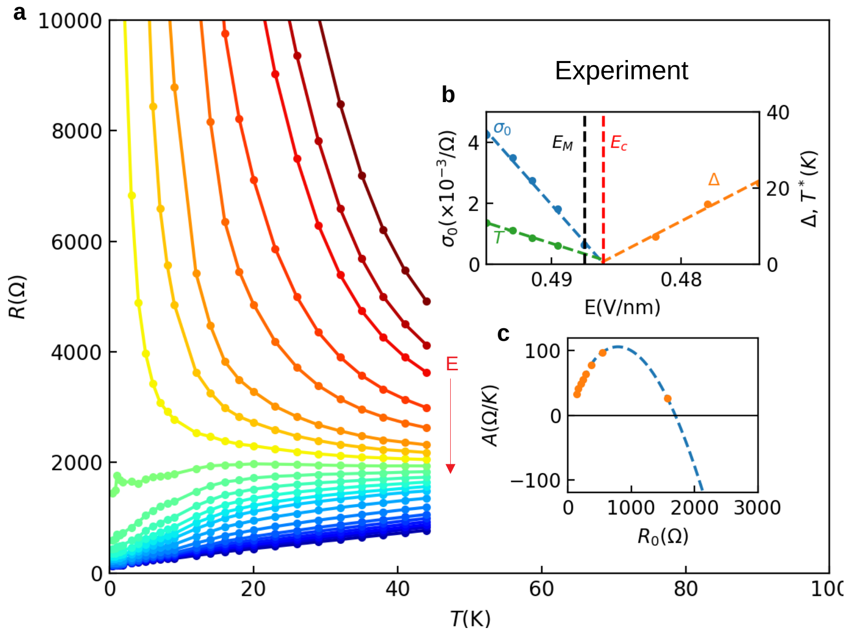

Supplementary Figure 6: Analyses for data obtained from device 1. **a** $R(T)$ curves reproduced from Ref. (*4*), with 0.4585V/nm $< E < 0.536$V/nm, where the dashed line corresponds to the critical curve; **b** three characteristic properties σ_0, T^* and $\Delta \sim \delta$ and **c** slope A on the metallic side.

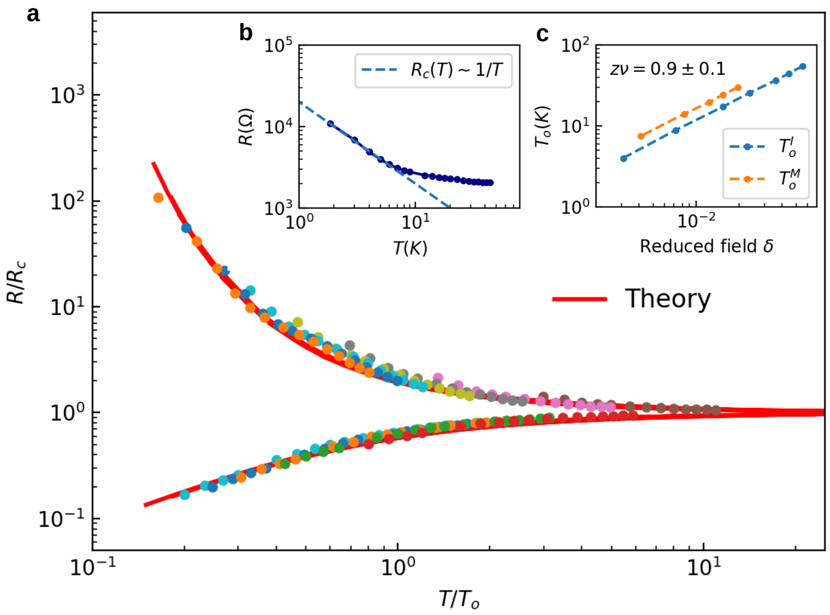

Supplementary Figure 7: Scaling analysis to data obtained from device 1. **a** Experimental data (dots) range: $(0.4585$V/nm $< E < 0.536$V/nm). **b** Critical exponent for resistivity $x = 1$, and **c** $z\nu \approx 1$.

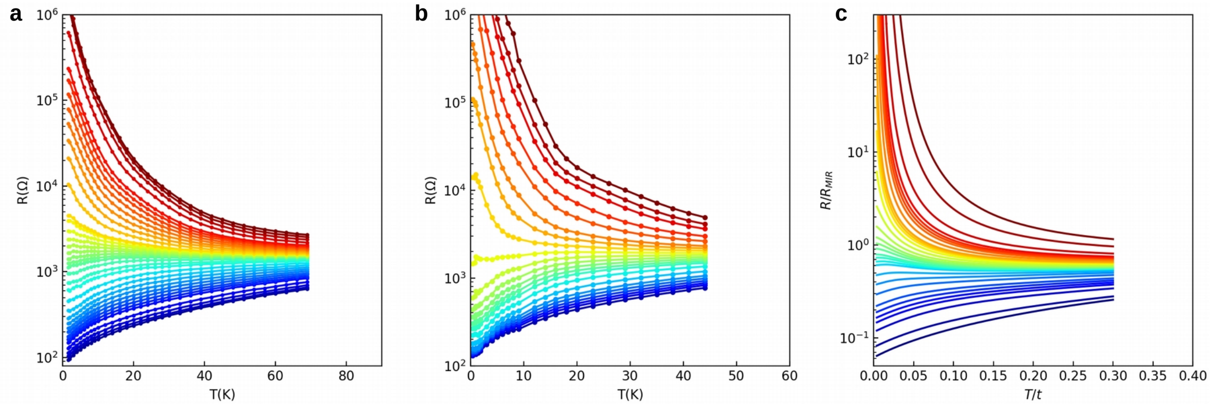

Supplementary Figure 8: Comparison of resistance curves on Log-Linear scale: **a** data obtained from device 2, **b** data obtained from device 1, **c** theoretical results.

which is comparable. Since strong disorder corresponds to $R_{highT} \sim R_{lowT}$, we conclude that both devices have a moderate amount of (bare) disorder, at a similar level.

Supplementary Note 5. Scaling procedures

In this section, we are providing the details of the scaling analysis. Firstly, as shown in Supplementary Fig. 9, the theoretical critical resistance $R_c \sim T^{-1}$. We then normalize the theoretical curves $R(T, \delta)$ by the critical resistance $R_c(T)$, and after re-scaling T by a field-dependent factor $T_o(\delta)$ (given in Fig. 4), the curves collapse onto two branches (red lines in Fig. 3). This "unbiased" procedure gives $c * T_o(\delta)$ up to an uncertain factor c, which just shift the branches horizontally. This factor then can be determined by making sure the two asymptotic forms of the scaling function $f(y)$ cross at $y = T/T_o(\delta) = 1$. Specifically, the metallic branch of $f(y)$ has two asymptotic forms: $f(y) \sim y$ when $y \sim 0$; $f(y) \to 1$ when $y \to \infty$. By forcing $T/T_o(\delta) = 1$, the position of the metallic branch on x-axis is fixed, so is the absolute value of $T_o(\delta)$ and the factor c. On the other hand, because the scaling function is a smooth function, the slopes of $T_o(\delta)$ on the metallic side and insulating side have to be the same, which also fix the position of the insulating branch on the x-axis. By now, the scaling analyses for theoreti-

cal results are complete. We then perform an identical scaling procedure on the experimental data. The theoretical and experimental branches collapse right on top of each other, giving remarkably the same scaling function $f(y)$ and the same critical exponent $z\nu = 1$.

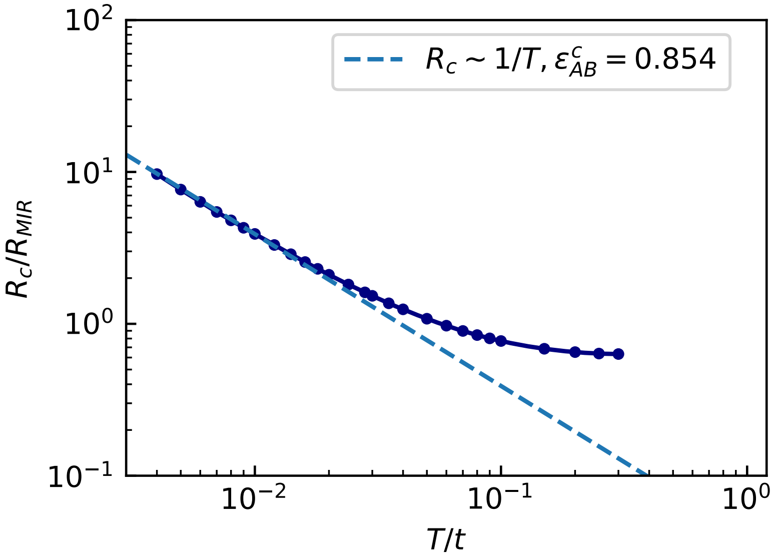

Supplementary Figure 9: The theoretical critical resistance R_c obtained at ϵ^c_{AB}, which is $\sim T^{-1}$.

Supplementary Note 6. Scaling analysis in a broad region

As mentioned in the main text, the scaling behavior should only be expected within the critical region, which is proven to not intersect with the Mooij point, even though the violation of scaling is observed to be small in the experimental results. Here for comparison, we also perform the scaling analysis to a broad region in theoretical results (Supplementary Fig. 10). The dots correspond to the results outside the critical region, which are put to collapse to the scaling function (red line) as good as possible. Firstly, on the metallic side, we can clearly see that the most metallic curve (e.g. orange dots) does not follow the scaling function, while the collapsing of others seems to be reasonably well, depending on how far they are close to the critical region.

Second, we can observe the violation of scaling more clearly on the metallic branch, while the collapsing is still perfect on the insulating side, simply because in our model the activation gap Δ is the only characteristic scale on the insulating side.

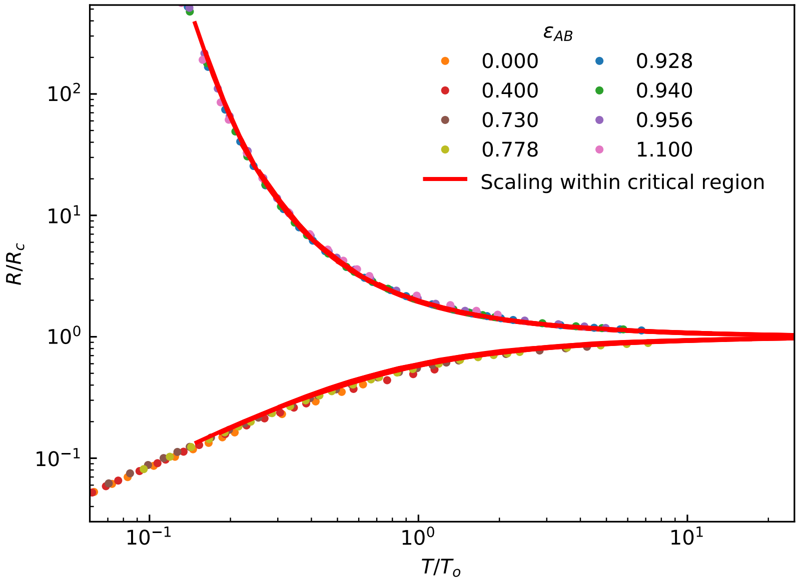

Supplementary Figure 10: Scaling analysis to broad region in theoretical results.

Supplementary Note 7. Degree of mirror symmetry of the scaling function

In this section, we discuss the concept of ”mirror symmetry” of the resistivity scaling function, which we find to be much less developed in the present disorder-dominated regime, than that in previously studied Mott-like situations. After one performs the scaling analysis as described in the main text, all the resistivity curves collapse on the two branches of the scaling function $f(y) = R/R_c$, with $y = T/T_o$. To provide a quantitative measure of the relative symmetry/asymmetry of the two branches (the metallic branch $f_M(y)$ and the insulating branch $f_I(y)$), we introduce the Mirror Symmetry Index (MSI), which can be defined as:

$$\mathrm{MSI} = f_I(y^*)/f_M(y^*), \tag{S15}$$

where y^* is the position above which $f_I = 1/f_M$. This simply means that when plotted on

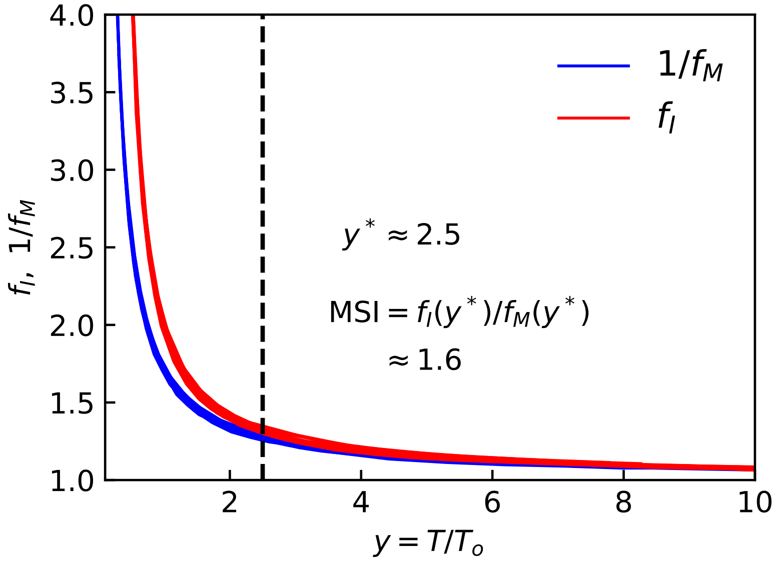

Supplementary Figure 11: The MSI here is pretty small, compared to MSI≈10 in the Mott case. f_I, f_M are scaling functions for metallic and insulating branches correspondingly. y^* is the position above which $f_I = 1/f_M$.

a logarithmic scale (see Supplementary Fig. 10), f_I and f_M display mirror symmetry about the horizontal line $f = 1$ (i.e. $R = R_c$), for $y > y^*$. As shown in Supplementary Fig. 11, the blue curve ($1/f_M$) and the red curve (f_I) collapse onto each other above y^*, which means that they display mirror symmetry, while they start to differ from each other below y^*. The corresponding value of MSI is just around 1.6, which is much smaller than what is found in the Mott regime ($f = 1$) in the same devices, as reported in Ref. (*4*), and also discussed in the recent paper by Jiawei Zang *et al.* (*5*). We would like to emphasize that smallness of MSI in the disordered case is not surprising, since here the temperature dependence of the resistivity on the metallic side is generally much weaker than on the insulating side (which displays activated transport behavior). This is in dramatic contrast to the Mott regime, where a very pronounced resistivity drop is found on the metallic side at low temperatures. Physically,

this reflects the thermal formation/destruction of heavy quasiparticles, an effect which is well established both within the DMFT-type theories (*6*), and by various experiments in the Mott regime. Consequently, MSI is much larger in the Mott regime, as one of the authors theoretically predicted in 2011 (*7*), and which was later confirmed by a beautiful series of experiments by K. Kanoda in Mott organics (*8*). The new data provided in Ref. (*4*) on TMD moiré hetero-bilayers are fully consistent with this early prediction concerning the Mott regime ($f = 1$), but not in the disorder-dominated regime ($f = 2$) we consider here.

Supplementary References

1. S. Ciuchi, D. D. Sante, V. Dobrosavljević, S. Fratini, The origin of Mooij correlations in disordered metals. *npj Quantum Materials* **3**, 1–6 (2018).

2. A. J. Millis, R. Mueller, B. I. Shraiman, Fermi-liquid-to-polaron crossover. I. General results. *Phys. Rev. B* **54**, 5389 (1996).

3. E. N. Economou, *Green's Functions in Quantum Physics* (Springer, Germany, 2005).

4. T. Li, *et al.*, Continuous Mott transition in semiconductor moiré superlattices, *Nature* **597**, 350–354 (2021).

5. J. Zang, J. Wang, J. Cano, A. Georges, A. J. Millis, Dynamical mean-field theory of moiré bilayer transition metal dichalcogenides: Phase diagram, resistivity, and quantum criticality. *Phys. Rev. X* **12**, 021064 (2022).

6. M. Radonjić, D. Tanasković, V. Dobrosavljević, K. Haule, Influence of disorder on incoherent transport near the Mott transition. *Phys. Rev. B* **81**, 075118 (2010).

7. H. Terletska, J. Vučičević, D. Tanasković, V. Dobrosavljević, Quantum critical transport near the Mott transition. *Phys. Rev. Lett.* **107**, 026401 (2011).

8. T. Furukawa, K. Miyagawa, H. Taniguchi, R. Kato, K. Kanoda, Quantum criticality of Mott transition in organic materials. *Nature Physics* **11**, 221–224 (2015).
